# Cloning and Characterization of *IbDREB1d* and Its Role in Plant Growth Regulation in Sweet Potato

**DOI:** 10.3390/plants15071135

**Published:** 2026-04-07

**Authors:** Guoliang Li, Yongqing Xu, Zhaomiao Lin, Hong Zhang, Sai Xie, Yongxiang Qiu, Guochun Xu, Huawei Li, Rongchang Ji, Wenbin Luo, Hao Tang, Si-Xin Qiu

**Affiliations:** Institute of Crop Sciences, Fujian Academy of Agricultural Sciences (Fujian Germplasm Resources Center), Fuzhou 350013, China; uslgl@126.com (G.L.); xuyongqing@faas.cn (Y.X.); linzhaomiao@faas.cn (Z.L.); zhanghong@faas.cn (H.Z.); xiesai@faas.cn (S.X.); qiuyongxiang@faas.cn (Y.Q.); xuguochuan@faas.cn (G.X.); lihuawei@faas.cn (H.L.); jirongchang@faas.cn (R.J.); luowenbin@faas.cn (W.L.); tanghao@faas.cn (H.T.)

**Keywords:** sweet potato, *IbDREB1d*, gene cloning, functional analysis, dwarf

## Abstract

DREB (Dehydration-Responsive Element-Binding Protein) transcription factors are a subfamily of the AP2/ERF transcription factor family and play a crucial role in the regulation of plant responses to abiotic stress. In this study, we successfully cloned the *IbDREB1d* gene from the leafy sweet potato cultivar Fucaishu18. The open reading frame (ORF) of the *IbDREB1d* gene comprises 792 base pairs and encodes a protein consisting of 263 amino acids. Protein sequence analysis indicates that IbDREB1d is characterized by acidic, hydrophilic, and unstable properties, with its closest phylogenetic relationships to *Ipomoea trifida* and *Ipomoea triloba*. Quantitative real-time PCR (RT-qPCR) analysis revealed that *IbDREB1d* is expressed in the roots, stems, and leaves of sweet potato, with increased expression under low temperature, hydrogen peroxide (H_2_O_2_), and drought conditions. Overexpression of *IbDREB1d* in sweet potato resulted in transgenic plants exhibiting dwarfism, shortened internode lengths, smaller leaf size, and microscopic evidence of impaired vascular tissue development. Hormonal analysis indicated significant reductions in the levels of indole-3-acetic acid, indole-3-butyric acid, salicylic acid, and zeatin in these transgenic plants. These decreases may explain the observed phenotypic changes, such as inhibited growth and reduced leaf size. This study provides novel theoretical insights into the role of *IbDREB1d* in stress-responsive expression and modulating plant growth in sweet potato.

## 1. Introduction

Abiotic stresses, such as drought, low temperature, and salinity, are key environmental factors that constrain crop growth and development and affect yield and quality [1,2]. To cope with these adversities, plants have evolved a complex and sophisticated molecular regulatory network over the long term. Within this network, transcription factors act as central hubs, playing a crucial role in plant stress tolerance by activating or repressing the expression of a series of downstream functional genes [3,4]. DREB (Dehydration-Responsive Element-Binding Protein) transcription factors belong to a subfamily of the AP2/ERF family and play a significant role in regulating plant responses to stress [5,6]. DREBs can specifically bind to the DRE/CRT cis-acting elements (A/GCCGAC) in the promoter regions of downstream target genes, thereby activating the expression of various stress-related genes (such as those involved in osmolyte biosynthesis, antioxidant enzyme systems, and protective proteins). They are key regulatory factors in the signaling pathways of plant responses to abiotic stress [7,8].

To date, *DREB* genes have been extensively identified and characterized in various plants, including Arabidopsis [9,10,11], rice [12,13,14,15,16], wheat [17,18,19], and soybean [20,21,22]. Liu et al. [9] discovered three transcription factors binding to CRT/DRE elements in cold-treated Arabidopsis, named DREB1A, DREB1B, and DREB1C. Subsequently, CBF4/DREB1D was identified in Arabidopsis; although it shares high homology with DREB1A, DREB1B, and DREB1C, it is induced by drought rather than low temperature [10]. Arabidopsis DDF1 and DDF2, which are members of the DREB subfamily also known as DREB1E and DREB1F, are primarily induced by high-salinity stress [11]. *OsDREB1A/B* are the earliest and most extensively studied rice CBF genes; overexpression of these genes significantly enhances the cold tolerance of transgenic rice [13]. In *OsDREB1G*-overexpressing rice, cold-responsive genes were highly induced compared to the wild type, but drought or salt tolerance was not significantly improved [14]. Thus, *DREB* genes have been proven to significantly improve plant drought, cold, and salt tolerance. Furthermore, some *DREB* genes can affect crop growth and development, making them key genes for crop yield [12,18].

Sweet potato (*Ipomoea batatas* L.) is a major food, feed, and industrial raw material crop in China. However, its production is often severely constrained by pests, weeds, and abiotic stresses [23]. He et al. [7] conducted a bioinformatics analysis of the AP/ERF gene family from the sweet potato ‘Taizhong 6’ genome, providing a reference for studying the function of sweet potato AP/ERF genes. Zhao [24], through transcriptome sequencing analysis of sweet potato, found that the transcription factor gene *DREB1B* was induced by low temperature, and its expression level was positively correlated with the activities of various antioxidant enzymes. Although the functions of *DREB* genes in other crops are relatively well understood, research regarding their role in sweet potato growth, development, and stress regulation, particularly their potential impact on important agronomic traits, remains relatively limited, and their molecular mechanisms remain to be further elucidated.

In this study, the vegetable-type sweet potato cultivar ‘Fucaishu 18’ was used as the material to clone the *IbDREB1d* gene and conduct bioinformatics analysis. We investigated its tissue expression characteristics and response patterns to different stress conditions. Transgenic plants were obtained through genetic transformation to deeply explore the biological function of this gene in sweet potato growth, development, and stress physiology. This study provides a new theoretical basis for elucidating the regulatory mechanism of DREB transcription factors in sweet potato and offers valuable candidate genes for improving sweet potato stress tolerance and plant architecture using genetic engineering approaches.

## 2. Materials and Methods

### 2.1. Plant Materials

The leafy sweet potato cultivar ‘Fucaishu 18’ was developed through hybridization and breeding by the Institute of Crop Sciences, Fujian Academy of Agricultural Sciences. It was used for the cloning and genetic transformation of the *IbDREB1d* gene to characterize its function.

### 2.2. Cloning and Bioinformatics Analysis Methods

Leaves from normally growing ‘Fucaishu 18’ plants in a greenhouse were used as the material. Total RNA was extracted using the FastPure Universal Plant Total RNA Isolation Kit (Vazyme Biotech Co., Ltd., Nanjing, China). Subsequently, approximately 1 μg of total RNA was reverse-transcribed into cDNA using the HiScript II 1st Strand cDNA Synthesis Kit (Vazyme Biotech Co., Ltd., Nanjing, China) to serve as the template for gene cloning. The reference CDS of IbDREB1d was retrieved from the Sweet Potato Genome Database (https://www.sweetpotato.com/, accessed on 15 August 2021). Homologous cloning primers were designed using Primer Premier 5 software (Table 1). The obtained CDS was translated into protein using Primer Premier 5 software. Conserved domain analysis was performed using the NCBI website. Multiple sequence alignment and phylogenetic analysis were conducted using Lasergene and MEGA15 software. Protein molecular weight and theoretical isoelectric point (pI) were predicted using Expasy (http://www.expasy.org, accessed on 12 October 2021).

### 2.3. Expression Analysis of the IbDREB1d Gene

After ‘Fucaishu 18’ seedlings were inserted into substrate blocks and cultivated for one month, they were subjected to treatments of low temperature (10 °C), 2.5 mmol L^−1^ H_2_O_2_, 20% PEG-6000, and 0.3% NaCl, respectively. Samples were collected at 0, 3, 6, 9, and 24 h after treatment. Roots, stems, and leaves of one-month-old tissue-cultured ‘Fucaishu 18’ seedlings were harvested, ground, and used for RNA extraction and reverse transcription to synthesize cDNA. Using cDNA as a template, RT-qPCR was performed using the Taq Pro Universal SYBR qPCR Master Mix kit (Vazyme Biotech Co., Ltd., Nanjing, China) to analyze the expression of the IbDREB1d gene. The sweet potato translation elongation factor EF1α was used as the reference gene [25]. The primer sequences used in the experiment are listed in Table 1.

### 2.4. Obtainment of Transgenic Sweet Potato Plants

The overexpression vector pEGOEP35S-G418-DREB1d was constructed using the coding sequence (CDS) of the *IbDREB1d* gene. The *IbDREB1d* CDS was amplified by PCR using sweet potato cDNA as a template. Specific primers were designed with *EcoRI* and *HindIII* restriction sites added to the 5′ and 3′ ends, respectively. The PCR product was digested with *EcoRI* and *HindIII* and ligated into the binary vector pEGOEP35S-G418, which had been linearized with the same enzymes. The resulting construct, pEGOEP35S-G418-DREB1d, contains a T-DNA region flanked by the Left Border (LB) and Right Border (RB) of Agrobacterium tumefaciens. Within the T-DNA, the IbDREB1d coding sequence is driven by the Cauliflower Mosaic Virus 35S (CaMV 35S) promoter and terminated by the nopaline synthase (NOS) terminator. The plant selectable marker gene, nptII, is located within the T-DNA region under the control of the NOS promoter. Positive clones were confirmed by colony PCR and Sanger sequencing (Supplementary Figure S1). The obtained recombinant vector was transformed into *Agrobacterium rhizogenes* strain K599. A single *Agrobacterium* colony was picked and cultured in 5 mL of LB medium containing antibiotics (kanamycin + streptomycin) at 28 °C with shaking for 1 day (OD600 > 0.4). Then, 0.5 mL of the bacterial culture was transferred into 30 mL of LB medium containing antibiotics and 0.5% glucose, and cultured at 28 °C with shaking for 16–18 h. The bacterial culture was centrifuged at 5000× *g* for 10 min at 4 °C, the supernatant was discarded, and the pellet was resuspended in 30 mL of 1/2 MS medium supplemented with 0.5% glucose and 0.1 mmol L^−1^ acetosyringone (AS). The suspension was cultured at 28 °C with shaking for 6 h to activate the infection capability of the bacteria (adjusted to OD600 = 0.2). Leaves were immersed in the activated bacterial suspension and infected with shaking at 125 r min^−1^ for 10–20 min. Subsequently, the leaves were washed several times with sterile water and dried with filter paper. The leaves (with cut ends touching the medium) were placed on 1/2 MS medium supplemented with 3% sucrose, 0.7% agar, and 0.01 mmol L^−1^ AS, and cultured in the dark for 3 days. The leaves were then transferred to 1/2 MS medium containing 125 mg L^−1^ Timentin, 125 mg L^−1^ cefotaxime, and 50 mg L^−1^ kanamycin, supplemented with 3% sucrose and 0.7% agar, for root induction. After rooting, the root systems were transferred to 1/2 MS medium containing 200 mg L^−1^ Timentin and 200 mg L^−1^ cefotaxime, supplemented with 3% sucrose and 0.7% agar, to induce shoot formation. When the shoots grew 3 to 5 leaves, they were transferred to MS medium for seedling development. The obtained putative transgenic plants were identified at the DNA level by PCR and at the RNA level by RT-qPCR. The primer sequences used in the experiment are listed in Table 1.

### 2.5. Observation of Biological Traits of Transgenic Sweet Potato Plants

The *IbDREB1d* transgenic sweet potato plants and wild-type control plants were transplanted from tissue culture seedlings to pots containing substrate and cultivated for one month. Plant growth vigor was observed, and stem and leaf lengths were measured. The third internode from the apex of both transgenic and wild-type plants was sampled for tissue sectioning and electron microscopy scanning. Tissue sectioning was commissioned to Wuhan Servicebio Technology Co., Ltd. (Wuhan, China) and electron microscopy scanning was performed at the Electron Microscopy Center of the Institute of Agricultural Quality Standards and Detection Technology, Fujian Academy of Agricultural Sciences.

### 2.6. Determination of Hormone Content in Transgenic Plant

Apical samples of 100 mg each were taken from the top three stem segments below the apex of transgenic plants and wild-type plants, respectively, and placed into centrifuge tubes. Then, 1 mL of pre-cooled 50% aqueous acetonitrile solution was added. The mixture was sonicated for 3 min at 4 °C and then left to extract statically for 30 min. After centrifugation at 10,000× *g* for 10 min, the supernatant was collected and passed through an RPSPE column. Next, 1 mL of 100% methanol solution and 1 mL of deionized water were added separately, and the column was equilibrated with 50% aqueous acetonitrile solution. After loading the sample, the column was washed with 1 mL of 30% aqueous acetonitrile solution, and the fraction was collected. The collected fraction was evaporated to dryness under a stream of nitrogen, reconstituted with 0.2 mL of 30% aqueous acetonitrile solution, and transferred to sample vials equipped with inserts for instrument analysis.

#### 2.6.1. Instrument and Parameters

The data acquisition instrument system mainly included an Ultra-High Performance Liquid Chromatography (Vanquish, UPLC, Thermo Fisher Scientific, Waltham, MA, USA) and a High-Resolution Mass Spectrometer (Q Exactive, Thermo Fisher Scientific, Waltham, MA, USA).

#### 2.6.2. Liquid Chromatography Parameters

Column: Waters ACQUITY UPLC HSS T3 column (50 mm × 2.1 mm, 1.8 μm) (Waters Corporation, Milford, MA, USA); Mobile phase: Phase A was ultrapure water solution (containing 0.1% acetic acid), Phase B was acetonitrile solution (containing 0.1% acetic acid); Flow rate: 0.3 mL min^−1^; Column temperature: 40 °C; Injection volume: 2 μg mL^−1^; Elution gradient: 0 min Phase A/Phase B (85:15, *v*/*v*), 0.5 min Phase A/Phase B (85:15, *v*/*v*), 1.5 min Phase A/Phase B (10:90, *v*/*v*), 3 min Phase A/Phase B (10:90, *v*/*v*), 3.1 min Phase A/Phase B (85:15, *v*/*v*), 5 min Phase A/Phase B (85:15, *v*/*v*). Throughout the analysis process, samples were placed in a 4 °C autosampler.

#### 2.6.3. Q Exactive High-Resolution Mass Spectrometry Detection System

Parameters: Equipped with an electrospray ionization (ESI) source; Sheath gas: 40 arb; Aux gas: 10 arb; Ion spray voltage: −2800 V/+3000 V; Temperature: 350 °C; Ion transfer tube temperature: 320 °C. The scanning mode was Single-Ion Detection Mode, and the scanning polarity was negative/positive ion. The primary mass spectrometry scanning range was 50–550.

### 2.7. Statistical Analysis

All experiments were performed in triplicate. Microsoft Excel 2021 was used to organize the experimental data and generate graphs. Statistical analysis was conducted using SPSS 27.0.1 software with Student’s *t*-test. Different lowercase letters indicate significant differences at the *p* < 0.05 level.

## 3. Results and Analysis

### 3.1. Cloning and Sequence Analysis of the IbDREB1d Gene

**Figure 1:**
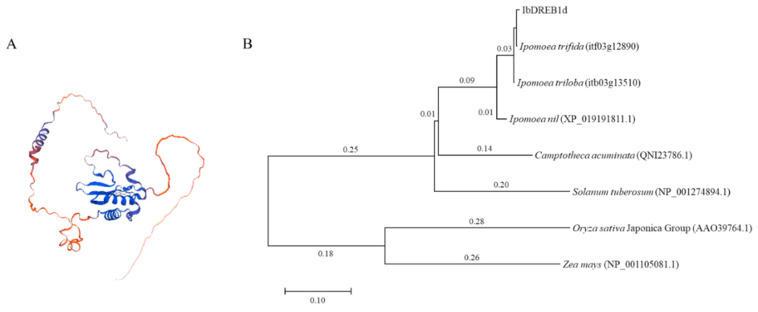
Bioinformatic analysis of IbDREB1d. (**A**) Tertiary structure analysis of IbDREB1d protein. (**B**) Homologous evolutionary tree analysis of IbDREB1d protein and related proteins in other species.

The CDS of the *IbDREB1d* gene was amplified from ‘Fucaishu 18’ (NCBI Accession No. OR188709.1). The full length is 792 bp, encoding 263 amino acids (aa). The predicted molecular weight is 28.95 kDa, the theoretical isoelectric point (pI) is 4.79, the instability index is 55.80, and the average hydrophobicity index is −0.392, indicating that the protein is acidic, hydrophilic, and unstable. SOPMA analysis indicated that the IbDREB1d protein consists of alpha-helices, extended strands, and random coils, accounting for 23.19%, 7.60%, and 69.20%, respectively. Figure 1A displays the three-dimensional structure of the IbDREB1d protein simulated by software. Phylogenetic tree analysis revealed that *IbDREB1d* is most closely related to the diploid wild relatives of sweet potato, *Ipomoea trifida* and *Ipomoea triloba*, and is distantly related to monocotyledonous plants such as rice and maize (Figure 1B). NLStradamus predicted a strong nuclear localization signal (NLS) at the N-terminal region (amino acids 67–94) of IbDREB1d (Figure S1), suggesting its nuclear accumulation.

### 3.2. Analysis of IbDREB1d Expression Characteristics

**Figure 2:**
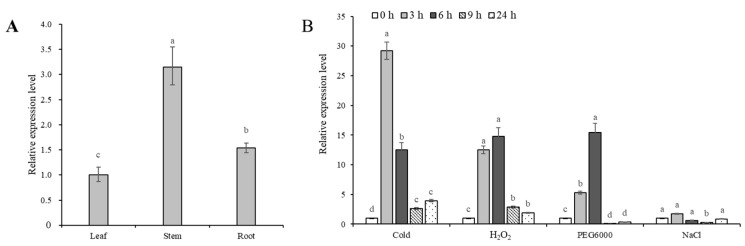
Expression analysis of *IbDREB1d* gene in Fucaishu 18. (**A**) Expression analysis of *IbDREB1d* in the roots (R), stems (S), and leaves (L) of Fucaishu 18. (**B**) Expression analysis of *IbDREB1d* in tissue-cultured seedlings of Fucaishu 18 under low-temperature, H_2_O_2_, 20% PEG6000, and NaCl treatments. Different lowercase letters indicate significant differences at *p* < 0.05.

RT-qPCR results showed that the *IbDREB1d* gene was expressed in the roots, stems, and leaves of ‘Fucaishu 18’, with the highest expression level in stems and the lowest in leaves (Figure 2A). Greenhouse seedlings of ‘Fucaishu 18’ were treated with 10 °C low temperature, 2.5 mmol L^−1^ H_2_O_2_, 20% PEG6000, and 0.3% NaCl. Using the 0 h stress treatment as the control, the expression level of *IbDREB1d* was set as 1. The expression of *IbDREB1d* was induced by low temperature, H_2_O_2_, and PEG6000, whereas induction by NaCl was not obvious. Among these, *IbDREB1d* expression increased most rapidly under low-temperature stress, increasing by 29-fold at 3 h and decreasing to 12-fold at 6 h. Under 2.5 mmol L^−1^ H_2_O_2_ treatment, the expression level of *IbDREB1d* increased by 12-fold compared to the control at 3 h and by 14-fold at 6 h. Under 20% PEG6000 stress, *IbDREB1d* expression increased by 5-fold at 3 h and by 15-fold at 6 h of treatment (Figure 2B).

### 3.3. Generation of IbDREB1d-Overexpressing Sweet Potato Plants

**Figure 3:**
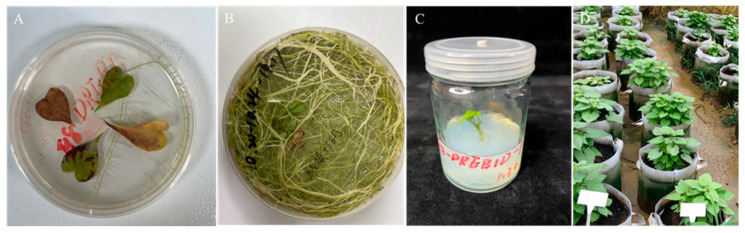
Production of transgenic sweet potato plants overexpressing the *IbDREB1d* gene. (**A**) Root induction of leaves from Fucaishu 18. (**B**) Bud differentiation induced from the roots of Fucaishu 18. (**C**) Cutting the bud to differentiate into a seedling. (**D**) Transplanting tissue-cultured seedlings into the field.

Leaves of ‘Fucaishu 18’ were inoculated with *Agrobacterium rhizogenes* harboring the recombinant overexpression vector containing the *IbDREB1d* gene (Figure S2). After a period of culture, numerous hairy roots formed at the base of the leaf veins. The roots were excised and subsequently cultured on 1/2 MS medium until adventitious buds differentiated, a process that typically required 4–6 months. The adventitious buds were cultured on MS medium to regenerate into complete plants (Figure 3).

**Figure 4:**
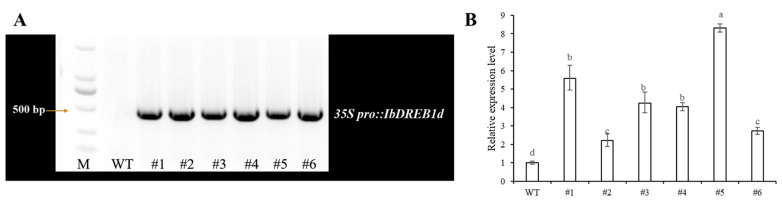
PCR amplification verification and RT-qPCR expression analysis of *IbDREB1d* gene in transgenic sweet potato plants. (**A**) Genomic PCR identification results of transgenic sweet potato plants. M: DNA marker; WT: Wild-type control; #1~#6: Different transgenic lines. (**B**) Relative expression levels of IbDREB1d gene in wild-type and transgenic plants detected by qRT-PCR. Different lowercase letters indicate significant differences at *p* < 0.05.

PCR analysis was performed to identify positive putative transgenic plants, and a total of six positive transgenic lines were obtained. Subsequently, RT-qPCR was performed to validate the transgenic lines. The results showed that the expression level of the *IbDREB1d* gene in the obtained transgenic lines was 2–8-fold higher than that in wild-type plants (Figure 4).

### 3.4. Transgenic Plants Exhibit Dwarfism and Reduced Leaf Size

**Figure 5:**
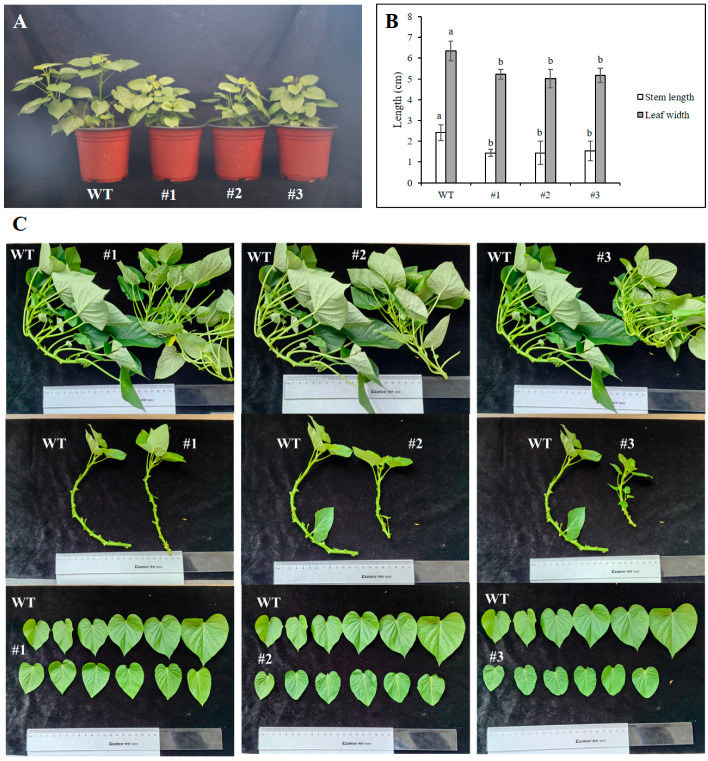
Identification of biological characteristics in sweet potato plants transgenic for *IbDREB1d* plants. (**A**) Transgenic and WT plants grown in pots for 1 month. (**B**) The stem length and leaf width of transgenic and WT plants. (**C**) Comparison of the size of stem sections and leaves between transgenic and WT. WT: wild type; #1–#3: transgenic lines. Different lowercase letters indicate significant differences at *p* < 0.05.

After the transgenic plants were propagated by cuttings in pots and grown for one month, it was observed that their growth was inferior to that of the wild-type ‘Fucaishu 18’, exhibiting a dwarf phenotype (Figure 5A). Comparison of their stem segment length and leaf size revealed that both the stem segment length and leaf width of the transgenic plants were smaller than those of the wild type. Specifically, the average stem segment length and leaf width of the transgenic plants were 1.44–1.53 cm and 5.02–5.23 cm, respectively, whereas those of the wild type were 2.45 cm and 6.35 cm, respectively (Figure 5B,C).

### 3.5. Microscopic Structure Observation of Transgenic Plants

**Figure 6:**
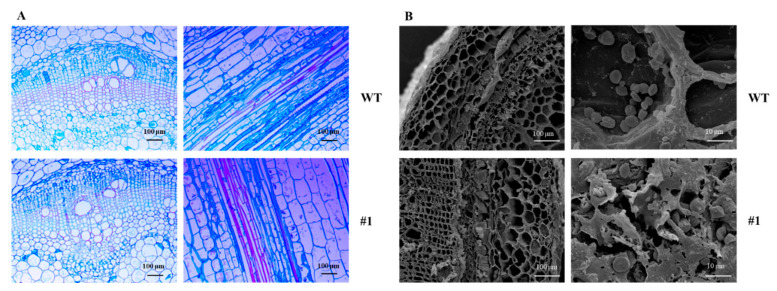
Comparison of transgenic plant and wild-type tissue sections. (**A**) Microscopic observation of paraffin sections of stem tissues (stained with safranin-fast green), (**B**) scanning electron microscopy observation of stem tissues. WT: wild type; #1: transgenic line.

Comparison of transverse tissue sections between transgenic and wild-type plants revealed that the vascular tissue in transgenic plants was smaller than that in the wild type, while the cell number remained unchanged. Comparison of longitudinal tissue sections showed no difference in cell length between the two, suggesting that the reduced stem segment size in transgenic plants may be attributed to a decrease in the number of cells along the longitudinal axis (Figure 6A). Scanning electron microscopy revealed that compared with the wild type, the size of starch grains in transgenic plants remained unchanged, but their number decreased (Figure 6B).

### 3.6. Comparative Analysis of Hormone Differences in Transgenic Plants

**Figure 7:**
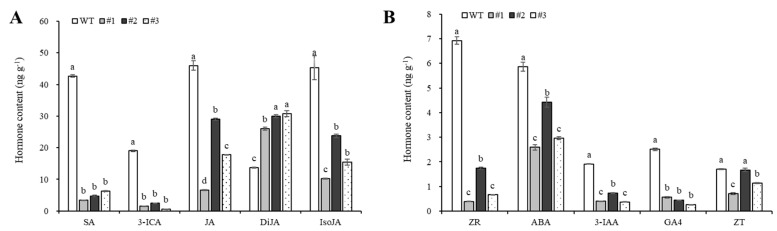
Comparison of apical hormone content between transgenic plants and wild-type plants. (**A**) Content analysis of hormones SA, JA, DiJA, IsoJA and 3-ICA. (**B**) Content analysis of hormones ZR, ABA, GA4, ZT and 3-IAA. SA: salicylic acid; 3-ICA: 3-indolecarboxylic acid; JA: jasmonic acid; DiJA: dihydrojasmonic acid; IsoJA: jasmonic acid-isoleucine; ZR: zeatin-riboside; ABA: abscisic acid; 3-IAA: indole-3-acetic acid; GA4: gibberellin A4; ZT: zeatin. WT: wild type; #1–#3: transgenic line. Different lowercase letters indicate significant differences at *p* < 0.05.

Comparison of hormone contents in the shoot tips of transgenic and wild-type plants revealed that the levels of most hormones were lower in transgenic plants than in the wild type, except for dihydrojasmonic acid, which increased. The decreases in salicylic acid and indole-3-carboxylic acid contents were particularly significant; salicylic acid content decreased from 42.69 ng g^−1^ to 3.43–6.33 ng g^−1^, and indole-3-carboxylic acid content decreased from 19.00 ng g^−1^ to 0.61–2.47 ng g^−1^. The contents of jasmonic acid and jasmonoyl-isoleucine varied significantly among transgenic plants, ranging from 6.57 to 29.07 ng g^−1^ and 10.21 to 23.75 ng g^−1^, respectively (Figure 7A). Additionally, the contents of zeatin riboside, zeatin, abscisic acid, indole-3-acetic acid, and GA4 were all decreased in the transgenic plants (Figure 7B). It is speculated that the hormones primarily responsible for the dwarf phenotype in transgenic plants include auxins such as indole-3-carboxylic acid and indole-3-acetic acid. Furthermore, the cold tolerance and drought resistance of the transgenic plants are inferior to those of the wild type.

## 4. Discussion

DREB transcription factors play a central role in the regulatory network governing plant responses to abiotic stresses such as drought, low temperature, high temperature, and salinity. Their AP2 domains exhibit highly conserved sequence and structural characteristics across different species [7]. In this study, the *IbDREB1d* gene was successfully cloned from the vegetable-type sweet potato cultivar ‘Fucaishu 18’, and its characteristics and functions were preliminarily explored. Sequence analysis indicated that the IbDREB1d protein possesses typical AP2 domain characteristics, providing clues for predicting its gene function (Figure S3). Phylogenetic analysis revealed that this protein is most closely related to the diploid wild relatives of sweet potato, *Ipomoea trifida* and *Ipomoea triloba*, which is consistent with the phylogenetic position of sweet potato within the Convolvulaceae family [26]. RT-qPCR expression analysis results showed that *IbDREB1d* is expressed in roots, stems, and leaves, suggesting it may play a role in the normal growth and development of sweet potato. The expression of this gene was also significantly induced by low temperature, H_2_O_2_ (reactive oxygen species signaling), and drought. This is highly consistent with the functions of known DREB family genes, confirming that *IbDREB1d* participates in the stress response pathway of sweet potato. It may regulate the cold and drought tolerance of sweet potato by controlling a series of stress-resistant genes containing DRE elements [6,18].

The most critical finding in this study is that overexpression of *IbDREB1d* resulted in severe developmental defects in transgenic plants, including dwarfism, shortened internodes, and reduced leaf size. This phenomenon is similar to previous findings by Kasuga et al., where transferring rice *DREB1A* into *Arabidopsis* and tobacco using the 35S and RD29A promoters also resulted in transgenic plants exhibiting growth inhibition and dwarfism phenotypes [27,28]. Similarly, overexpression of *OsDREB2B* caused dwarfism in transgenic rice plants. *OsDREB2B* can directly bind to and activate the expression of *GA2ox1*, a key gene in gibberellin metabolism, leading to the decomposition and inactivation of biologically active GA. This results in a decrease in active GA levels within the plant, triggering dwarfism [29]. Overexpression of *TaDREB2* and *TaDREB3* in barley and wheat also resulted in phenomena such as slow growth, reduced yield, and delayed flowering [30]. The use of inducible promoters such as *HDZI-3* and *HDZI-4* can mitigate the adverse effects of slow growth and yield reduction while improving crop stress tolerance [18]. Based on this, it is speculated that *IbDREB1d* not only participates in the stress response of sweet potato but also regulates vascular tissue development and cell division by affecting plant hormone synthesis or signal transduction, ultimately influencing sweet potato plant architecture and leaf size. This indicates that there is a delicate regulatory balance between stress resistance and growth development mediated by the *IbDREB1d* gene, and precise regulation of its expression level is crucial for establishing an ideal plant architecture in crops.

In summary, this study is the first to reveal the function of the IbDREB1d gene in sweet potato. This gene not only responds to abiotic stress but also regulates hormone changes and vascular tissue development to influence sweet potato plant architecture formation and leaf size. Future research should focus on utilizing techniques such as DNA Affinity Purification Sequencing (DAP-seq) and RNA-seq to identify the direct downstream target genes of IbDREB1d, particularly those key genes involved in hormone synthesis and signal transduction, thereby comprehensively elucidating its regulatory network. Furthermore, exploring the use of stress-inducible or tissue-specific promoters to drive IbDREB1d expression aims to achieve breakthroughs in cultivating new sweet potato germplasm with excellent plant architecture and high stress resistance.

## 5. Conclusions

In this study, the *IbDREB1d* gene was cloned from the vegetable-type sweet potato cultivar ‘Fucaishu 18’. The full-length CDS of this gene is 792 bp, encoding 263 amino acids. The *IbDREB1d* gene exhibited the highest expression level in sweet potato stem segments and could be induced by low temperature, H_2_O_2_, and PEG. Overexpression of the *IbDREB1d* gene in sweet potato resulted in a 2–8-fold increase in expression levels compared to wild-type plants. The transgenic plants exhibited dwarfism, shortened internodes, and reduced leaf size, accompanied by impaired vascular tissue development and decreased contents of multiple key hormones in the shoot tips. This study provides a new candidate gene for stress resistance breeding and plant architecture improvement in sweet potato.

## Figures and Tables

**Table 1:** Primers used in this study.

Primer Name	Primer Sequence (5′–3′)	Application of Primer
DREB1d-F	ATGGATTACTCGACTTCG	Cloning of the *DREB1d* gene
DREB1d-R	CTAGGTTCGCTCCTCACAAA	
pEGO35S-F	TGACATGATTACGAATTC	Detection of transgenic plants
pEGO35S-R	GGTGGCAAGAGTCCCCC	
qDREB1d-F	GTCTTCGCCACTGTCTTCTT	RT-qPCR of *DREB1d*
qDREB1d-R	GCGCGTTTCTTCGGATTATTAG	
qEF1α-F	TGCCTTGTGGAAGTTTGA	Reference genes for RT-qPCR
qEF1α-R	GGAGTATTTGGGAGTGGTG	

## Data Availability

The original contributions presented in this study are included in the article/Supplementary Material. Further inquiries can be directed to the corresponding author.

## Supplementary Materials

The following supporting information can be downloaded at: https://www.mdpi.com/article/10.3390/plants15071135/s1, Figure S1. Prediction of nuclear localization signal (NLS) in IbDREB1d; Figure S2. Plasmid map of the plant expression vector pEGOEP35S-G418-DREB1d. The circular map indicates the 10,329 bp sequence featuring the CaMV 35S promoter, the DREB1d gene, T-DNA borders, and antibiotic resistance markers; Figure S3. Multiple sequence alignment of IbDREB1d with homologous DREB1 proteins from other species.
