# Supplementary material for: Cloning and Characterization of IbDREB1d and Its Role in Plant Growth Regulation in Sweet Potato
Source: Plants (Basel). 2026 Apr 7;15(7):1135. doi: 10.3390/plants15071135 (PMC13075163; doi:10.3390/plants15071135)
Supplement: Supplementary file 1 [file plants-15-01135-s001.zip › plants-4198904-supplementary.pdf]

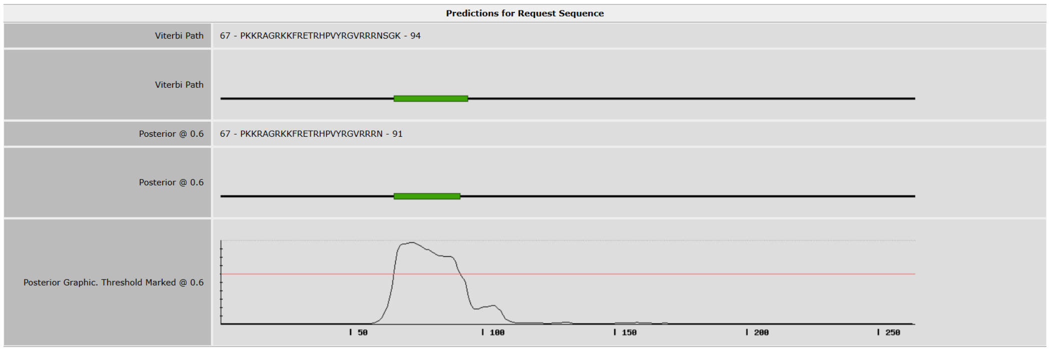

Figure S1. Prediction of nuclear localization signal (NLS) in IbDREB1d.

Notes:The amino acid sequence of IbDREB1d was analyzed using NLStradamus. A strong putative NLS motif was identified between amino acids 67 and 94 (highlighted in green), with a posterior probability significantly exceeding the threshold (0.6).

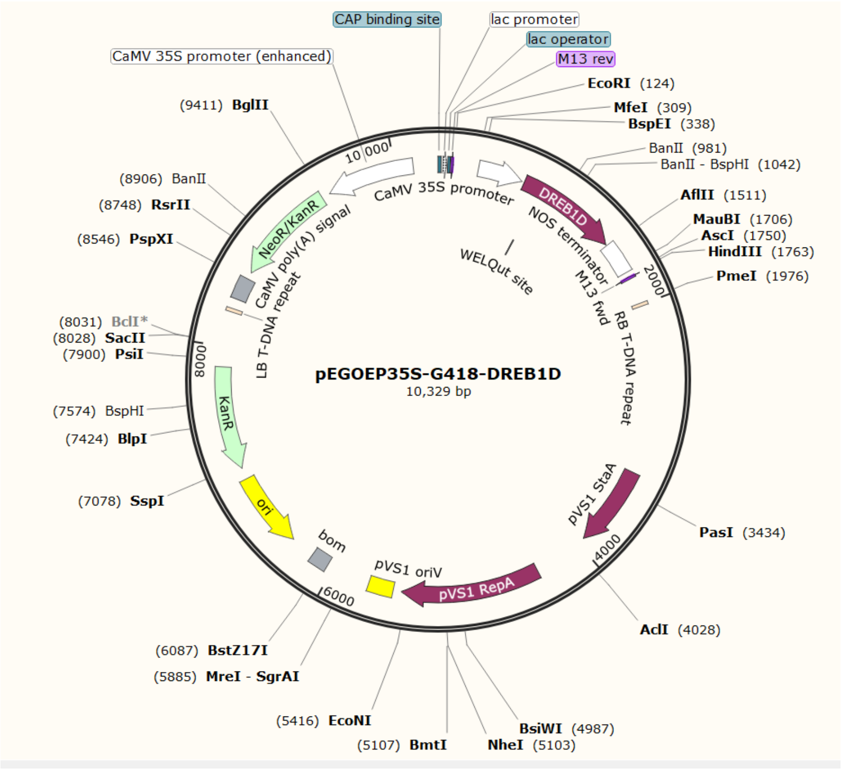

Figure S2. Plasmid map of the plant expression vector pEGOEP35S-G418-DREB1d. The circular map indicates the 10,329 bp sequence featuring the CaMV 35S promoter, the DREB1d gene, T-DNA borders, and antibiotic resistance markers.

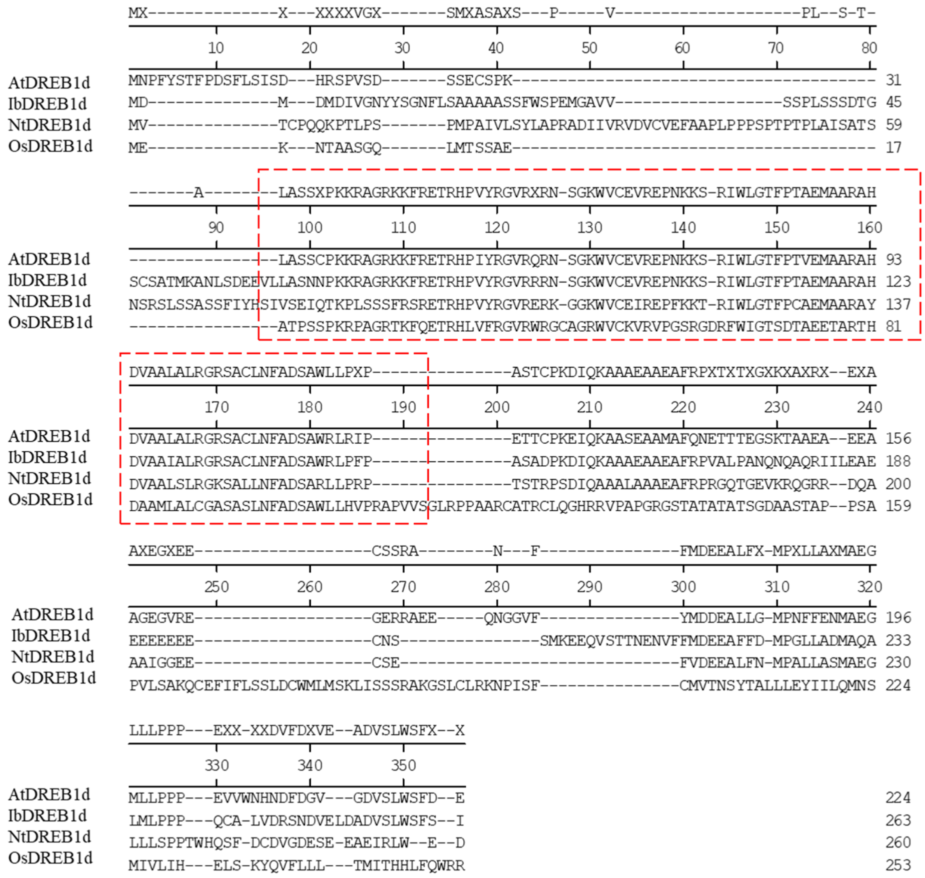

Figure S3. Multiple sequence alignment of IbDREB1d with homologous DREB1 proteins from other species.

The amino acid sequences of IbDREB1d and its homologs from *Arabidopsis thaliana* (AtDREB1d), *Nymphaea thermarum* (NtDREB1d), and *Oryza sativa* (OsDREB1d) were aligned using DNASTAR. The conserved AP2/ERF DNA-binding domain is indicated by red dashed boxes.
